# Noroviruses Co-opt the Function of Host Proteins VAPA and VAPB for Replication via a Phenylalanine–Phenylalanine-Acidic-Tract-Motif Mimic in Nonstructural Viral Protein NS1/2

**DOI:** 10.1128/mBio.00668-17

**Published:** 2017-07-11

**Authors:** Broc T. McCune, Wei Tang, Jia Lu, James B. Eaglesham, Lucy Thorne, Anne E. Mayer, Emily Condiff, Timothy J. Nice, Ian Goodfellow, Andrzej M. Krezel, Herbert W. Virgin

**Affiliations:** a Department of Pathology and Immunology, Washington University School of Medicine, St. Louis, Missouri, USA; b Department of Biochemistry and Molecular Biophysics, Washington University School of Medicine, St. Louis, Missouri, USA; c Department of Pathology, Division of Virology, University of Cambridge, Cambridge, United Kingdom; University of Pittsburgh School of Medicine

**Keywords:** noroviruses, plus-strand RNA virus, protein structure-function, reverse genetic analysis, viral replication, virus-host interactions

## Abstract

The *Norovirus* genus contains important human pathogens, but the role of host pathways in norovirus replication is largely unknown. Murine noroviruses provide the opportunity to study norovirus replication in cell culture and in small animals. The human norovirus nonstructural protein NS1/2 interacts with the host protein VAMP-associated protein A (VAPA), but the significance of the NS1/2-VAPA interaction is unexplored. Here we report decreased murine norovirus replication in VAPA- and VAPB-deficient cells. We characterized the role of VAPA in detail. VAPA was required for the efficiency of a step(s) in the viral replication cycle after entry of viral RNA into the cytoplasm but before the synthesis of viral minus-sense RNA. The interaction of VAPA with viral NS1/2 proteins is conserved between murine and human noroviruses. Murine norovirus NS1/2 directly bound the major sperm protein (MSP) domain of VAPA through its NS1 domain. Mutations within NS1 that disrupted interaction with VAPA inhibited viral replication. Structural analysis revealed that the viral NS1 domain contains a mimic of the phenylalanine–phenylalanine-acidic-tract (FFAT) motif that enables host proteins to bind to the VAPA MSP domain. The NS1/2-FFAT mimic region interacted with the VAPA-MSP domain in a manner similar to that seen with bona fide host FFAT motifs. Amino acids in the FFAT mimic region of the NS1 domain that are important for viral replication are highly conserved across murine norovirus strains. Thus, VAPA interaction with a norovirus protein that functionally mimics host FFAT motifs is important for murine norovirus replication.

## IMPORTANCE

Human noroviruses are a leading cause of gastroenteritis worldwide, but host factors involved in norovirus replication are incompletely understood. Murine noroviruses have been studied to define mechanisms of norovirus replication. Here we defined the importance of the interaction between the hitherto poorly studied NS1/2 norovirus protein and the VAPA host protein. The NS1/2-VAPA interaction is conserved between murine and human noroviruses and was important for early steps in murine norovirus replication. Using structure-function analysis, we found that NS1/2 contains a short sequence that molecularly mimics the FFAT motif that is found in multiple host proteins that bind VAPA. This represents to our knowledge the first example of functionally important mimicry of a host FFAT motif by a microbial protein.

## INTRODUCTION

Noroviruses (NoVs) are nonenveloped positive-sense single-stranded RNA viruses that primarily infect the gastrointestinal tract. They are a leading cause of gastroenteritis worldwide (1–3). Noroviruses are divided into genogroups GI to GVI. Among those genogroups, GI, GII, and GIV viruses cause human disease and GV encompasses more recently discovered rodent NoVs, including murine norovirus (MNoV) (4). As MNoVs replicate robustly in mice and cells and can be studied via mutagenesis of infectious molecular clones, they serve as a powerful model for molecular studies of norovirus replication, tropism, and pathogenesis (5, 6).

The norovirus genome encodes nine known proteins: seven nonstructural (NS) proteins derived by proteolysis of the open reading frame (ORF) 1 polyprotein (7) and two structural proteins, VP1 and VP2, derived from ORFs 2 and 3, respectively (6). MNoV encodes virulence protein VF1 from ORF 4, which overlaps ORF 2 and has not been found in human noroviruses (8). The N-terminal protein in the norovirus polyprotein, NS1/2, comprises three domains: NS1, NS2, and a putative transmembrane domain (9). The MNoV NS1 domain in isolation has a structured region preceded by an unstructured domain (9, 10). A single aspartic acid-to-glutamic acid difference within NS1 confers an altered conformation within the NS1 structured domain (10) and is associated with enteric tropism and the capacity of MNoV to persistently infect and be shed from the mouse intestine (11). Ectopically expressed NS1/2 from GI human norovirus (NS1/2^GI^) disrupts the Golgi apparatus and vesicular trafficking (12, 13) and is reported to interact with the host protein VAMP-associated protein A (VAPA) (12). The role of VAPA interactions with NS1/2 during viral replication has not been defined.

VAPA is a type II endoplasmic reticulum (ER)-resident protein that is conserved in eukaryotes (14). VAPB is structurally related to VAPA (15). VAPA comprises a major sperm protein (MSP) domain, a coiled-coil domain (CCD), and a transmembrane domain. Initially found to bind to proteins within the SNARE superfamily of vesicle trafficking proteins (16–18), VAPA also binds a variety of client interacting proteins (14). Importantly, through its cytosolic MSP domain, VAPA interacts with client proteins primarily involved in lipid trafficking (14, 19–23). These client proteins interact with the VAPA-MSP domain via a phenylalanine–phenylalanine-acidic-tract (FFAT) linear motif (22, 24–27).

VAPA performs important functions during infection as both microbes and antimicrobial host molecules target VAPA and its client proteins. VAPA and VAPB enhance the replication of hepatitis C virus (28, 29), rhinoviruses (30), tombusvirus (31, 32), and the intracellular bacteria *Chlamydia trachomatis* (33, 34). Some of these microbes encode molecules that interact with VAPA and VAPB and/or their client proteins, including hepatitis C virus proteins NS5a and NS5b (28, 29), tombusvirus p33 (31, 32), and *C. trachomatis* IncD (33, 34). Several observations support the idea that VAPA and VAPA client proteins assist in organization of membranous structures critical for virus replication (35, 36), possibly by manipulating the lipid composition of these membranes (30–32). Furthermore, VAPA binds to proteins regulated by interferon, interferon-induced transmembrane protein 3 (37), and viperin (38, 39), suggesting that VAPA may be involved in antiviral responses.

Here we found that VAPA enhances MNoV replication and defined the molecular basis of NS1/2-VAPA interactions. Disruption of VAPA in permissive cells delayed MNoV replication due to effects occurring after viral entry but prior to synthesis of viral minus-sense RNA. VAPB was also important for MNoV replication and bound MNoV NS1/2. The interaction between NS1/2 and VAPA was conserved between human norovirus and MNoV NS1/2 proteins. The NS1 domain of MNoV NS1/2 interacted with the MSP domain of VAPA. This interaction occurred independently of other cellular or viral proteins and mapped to a short region in the NS1 domain sharing features of the FFAT motif found in host proteins that also interact with the VAPA MSP domain. NS1 engaged VAPA MSP domain residues crucial for interaction with FFAT motifs found in VAPA client proteins. Mutagenesis of conserved amino acids in NS1 to abrogate VAPA interaction impaired recovery of infectious MNoV after transfection of permissive cells with plasmids encoding the viral genome. These data indicate that NS1/2-VAPA binding is critical for efficient MNoV replication and that this occurs through viral mimicry of the host FFAT motif by amino acids in the NS1 domain of the nonstructural NS1/2 protein.

## RESULTS

### Murine norovirus replication is diminished in VAPA-deficient cells.

**FIG 1:**
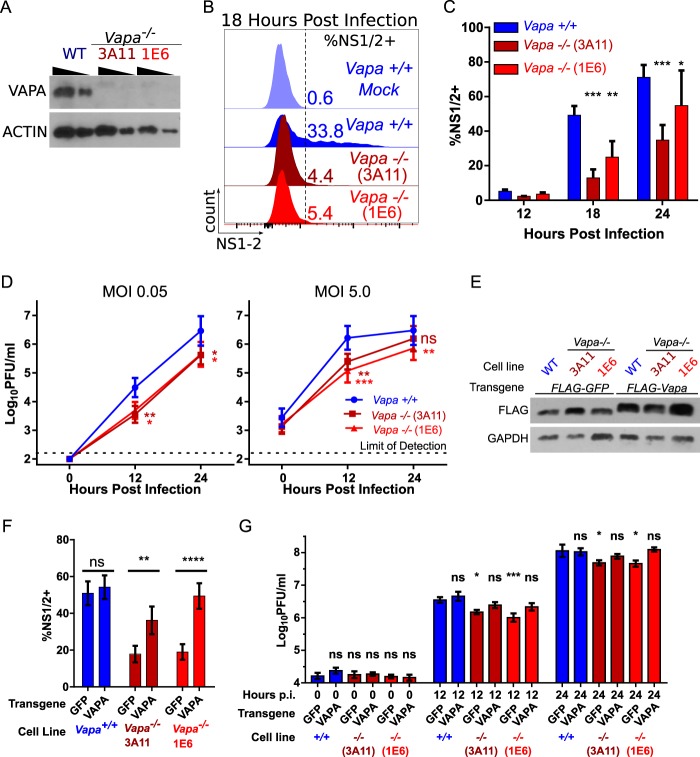
Murine norovirus replication in *Vapa*^−/−^ cells is diminished. (A) VAPA Western blot of *Vapa*^*−/−*^ cell lines. (B) Representative infection frequency of MNoV-CW3 in *Vapa*^*−/−*^ cells, measured by FACS analysis of intracellular NS1/2 (18 h postinfection; MOI of 5.0). (C) Same as panel B. Data represent results of combined experiments (repeated-measure two-way ANOVA, Dunnett posttest; *n* = 3). (D) MNoV strain CW3 growth in *Vapa*^*−/−*^ and *Vapa*^*+/+*^ cell lines (MOI, 0.05 [left] or 5.0 [right] PFU/cell). Data represent results of repeated-measure one-way ANOVA and the Dunnett posttest (*n* = 6). (E) Western blot of *Vapa*^*+/+*^ or *Vapa*^*−/−*^ cell lines lentivirally transduced with *FLAG-GFP* or *FLAG-Vapa*. (F) Infection frequency in *Vapa*- or *GFP*-transduced cells determined as described for panel B (two-way ANOVA, Sidak posttest; *n* = 9). (G) CW3 growth in *Vapa*- or *GFP*-transduced cells. Data represent results of repeated-measure two-way ANOVA and the Dunnett posttest (*n* = 5). For G the asterisks refer to a comparison to the time-matched +/+ GFP control.

To test the hypothesis that MNoV replication involves VAPA, we genetically engineered RAW 264.7 cells deficient in VAPA expression (here *Vapa*^*−/−*^) using clustered regularly interspaced short palindromic repeats (CRISPR)-Cas9. In two single-cell cloned *Vapa*^*−/−*^ cell lines, 3A11 and 1E6, frameshifts in the first 37 nucleotides (see Fig. S1A in the supplemental material) of coding sequence resulted in loss of VAPA protein expression (Fig. 1A). *Vapa*^−/−^ cells infected with MNoV strain CW3 had 2.2-fold-fewer (1E6) or 4.0-fold-fewer (3A11) NS1/2-positive cells by flow cytometry at 18 h postinfection (hpi) than wild-type (WT) cells (Fig. 1B and C). We observed lower levels of replication of MNoV strains CW3 and CR6 in both *Vapa*^*−/−*^ cell lines (Fig. 1D; Fig. S1B). Reconstituting VAPA production in *Vapa*^−/−^ cells via lentivirus transduction (Fig. 1E) increased the percentage of cells expressing NS1/2 at 18 h postinfection by 2.7-fold (3A11) or 4.1-fold (1E6) compared to transduction with green fluorescent protein (GFP) (Fig. 1F). Expression of VAPA, but not GFP, rescued viral replication in *Vapa*^−/−^ cells (Fig. 1G). Because VAPA deficiency incompletely blocked MNoV replication, we considered the possibility that VAPB might compensate for VAPA function. We found that VAPB was also important for MNoV replication (Fig. S1C). We were unable to efficiently isolate cell lines containing out-of-frame mutations in both VAPA and VAPB to directly test the possibility that these two proteins might compensate for each other (not shown). Furthermore, we were unable to test the role of VAPA in mice as mutation of *Vapa* led to embryonic lethality (Fig. S1D to F). We conclude that MNoV infectivity was enhanced by VAPA expression and chose to examine the mechanism responsible in more detail for VAPA.

10.1128/mBio.00668-17.1 FIG S1  Supplemental material corresponding to Fig. 1. (A) Alignment of genomic sequence and translation for two *Vapa* edited RAW 264.7 cell lines with wild-type *Vapa*^*+/+*^ (NM_013933) (top), showing both mutant alleles for each cell line. Numbering is relative to the transcript start. (B) MNoV strain CR6 growth in *Vapa*^*−/−*^ and *Vapa*^*+/+*^ cell lines (MOI, 0.05 [left] or 5.0 [right] PFU/cell). Repeated-measure one-way ANOVA and the Dunnett posttest were used; *n* = 6. (C) Infection frequency of MNoV-CW3 in BV2-Cas9 cells expressing an sgRNA (indicated on the *x* axis). Data were determined by intracellular FACS analysis of NS1/2 (18 hpi, MOI of 0.1). Analyses were performed using one-way ANOVA and the Dunnett posttest; *n* = 4 to 6. Data represent percent nonhomologous end joining (NHEJ) at sgRNA-targeted loci estimated using T7 endonuclease assay. (D) Alignment of two *Vapa* mutant mouse lines generated by electroporating embryonic stem cells with Cas9 and gRNA targeting *Vapa*. Note that mutant line 1 has its splice junction deleted, and mutant line 2 has a single-base-pair insertion. Numbering is relative to the transcript start for ease in comparing with panel A data. (E) Genotype of live pups from heterozygote crosses. (F) Genotype of day 14 embryos. Embryonic day 14 embryos (from four litters in total) were isolated from heterozygote crosses and genotyped. Undetermined, samples for which PCR amplification failed. Download FIG S1, EPS file, 0.9 MB. Copyright © 2017 McCune et al. 2017 McCune et al. This content is distributed under the terms of the Creative Commons Attribution 4.0 International license.

### VAPA is important for an early postentry step in norovirus replication.

**FIG 2:**
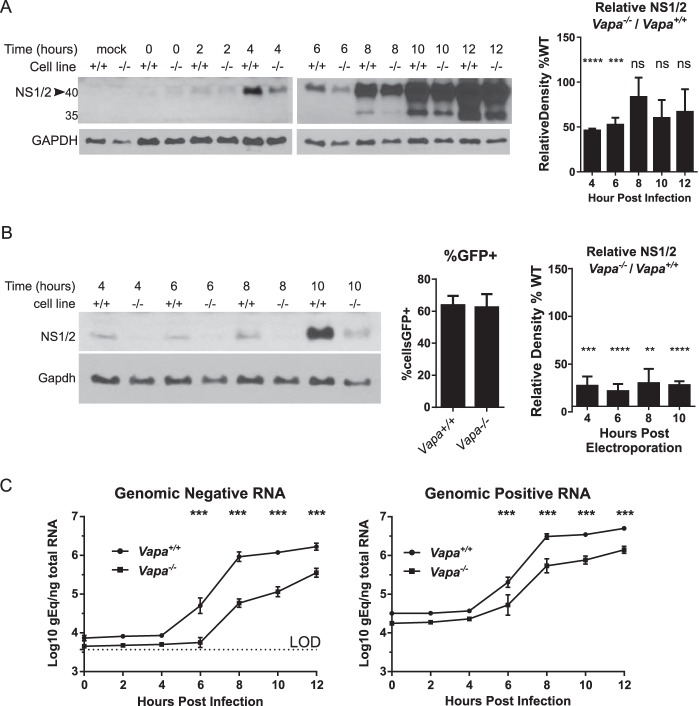
Murine norovirus replication in RAW 264.7-*Vapa*^*−/−*^ cells is impaired early in the viral life cycle. (A) Western blot of NS1/2 in *Vapa*^*+/+*^ and *Vapa*^*−/−*^ (3A11) cell lines (MOI of 5). (Right panel) Combined densitometry data from multiple experiments performed on film exposures for each time point within the linear range of assay (*n* = 2 to 4) (unpaired *t* test, means compared to H_*o*_ = 100). (B) NS1/2 Western blot after electroporation of viral RNA (vRNA) into *Vapa*^*+/+*^ and *Vapa*^*−/−*^ 3A11 cells (representative, *n* = 3 to 5). (Middle panel) *Vapa*^*+/+*^ and *Vapa*^*−/−*^ cells were transfected equivalently with pMAX-GFP. (Right panel) Combined densitometry data determined as described for panel A (*n* = 3 to 5). (C) Viral-strand-specific quantitative PCR for negative strand (left) and positive strand (right) over time in infected *Vapa*^*+/+*^ and *Vapa*^*−/−*^ 3A11 cells (MOI of 5; *n* = 3; two-way ANOVA).

To investigate the role of VAPA in MNoV replication, we analyzed nonstructural protein expression by assessing NS1/2 protein levels in infected cells by Western blotting. Infected *Vapa*^−/−^ cells expressed lower levels of NS1/2 protein at 4 and 6 hpi (Fig. 2A), with the difference diminishing later in infection. This supports the notion of a role for VAPA in early events of MNoV replication. Because VAPA is associated with efficient entry of an enveloped virus (37) as well as with the function of endosomes (19, 37, 40), through which MNoV likely passes to establish infection (41–43), we tested whether impaired viral entry in *Vapa*^*−/−*^ cells accounted for decreased NS1/2 production and viral replication. We reasoned that transfection of viral RNA would bypass any effect of VAPA on viral entry and uncoating. After electroporating purified viral RNA into cells, we continued to detect decreased NS1/2 levels in *Vapa*^−/−^ cells (Fig. 2B), despite observing no difference in transfectability as measured by plasmid-driven GFP expression (Fig. 2B, middle panel). These data indicate that VAPA plays a role in viral protein expression downstream of viral entry.

After the viral RNA accesses the cytoplasm, NS1/2 protein can be produced either by translation of virion-derived plus-sense viral RNA or by transcription of plus-sense viral RNA from newly synthesized minus-sense RNA. Using strand-specific reverse transcription-quantitative PCR (RT-PCR) (44), we observed delayed accumulation of both negative-sense and positive-sense MNoV RNA in the *Vapa*^*−/−*^ 3A11 (Fig. 2C) and 1E6 (Fig. S2) cell lines, indicating that production of NS1/2 is impaired prior to synthesis of new viral minus-sense RNA. Collectively, these observations support the notion of a role for VAPA downstream of viral RNA delivery into the cytosol but upstream of minus-sense viral RNA synthesis.

10.1128/mBio.00668-17.2 FIG S2  Supplemental material corresponding to Fig. 2. Viral-strand-specific quantitative PCR for the negative strand (left) and positive strand (right) over time in infected *Vapa*^*+/+*^ and *Vapa*^*−/−*^ (1E6) cells (MOI of 5; *n* = 3, two-way ANOVA, Sidak posttest). Download FIG S2, EPS file, 0.1 MB. Copyright © 2017 McCune et al. 2017 McCune et al. This content is distributed under the terms of the Creative Commons Attribution 4.0 International license.

### NS1/2 interaction with VAPA is conserved among norovirus strains.

**FIG 3:**
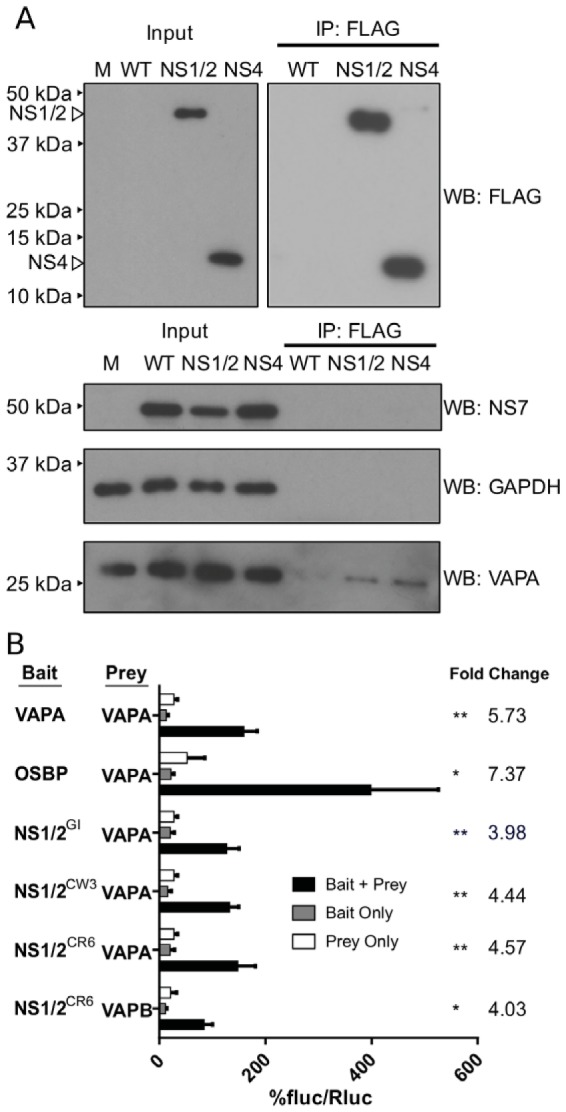
NS1/2 interactions with VAPA are conserved between norovirus strains and occur during infection. (A) BV2 cells were infected with NS1/2-FLAG or NS4-FLAG MNV for 8 h (MOI of 10 TCID_50_/cell). FLAG pulldown was performed on lysates, and immunoblotting was performed with the specified antibodies. M, molecular marker. (B) M2H interaction of NS1/2^GI^, NS1/2^MNoV^ (CR6 and CW3), OSBP, and VAPA with VAPA or VAPB (bottom) (one-way ANOVA, Dunnett posttest; fold change data are shown on the right; *n* = 3). fluc, firefly luciferase; Rluc, *Renilla* luciferase.

Prior work showed that VAPA interacts with GI human norovirus NS1/2 protein (NS1/2^GI^) (12). To test if VAPA interaction with NS1/2 is conserved across genogroups and species boundaries, we engineered MNoV to express a FLAG tag in NS1/2 (nucleotide 383) and also studied a previously described virus with a FLAG tag in NS4 (nucleotide 2600) (Fig. S3A) (45). We selected NS4 for this experiment as it is known to bind NS1/2 (45). Both MNoV-NS1/2^FLAG^ and MNoV-NS4^FLAG^ replicated similarly to wild-type virus (Fig. S3B). FLAG-tagged viral proteins of appropriate molecular mass were expressed during infection (Fig. 3A, top left). As expected, virus-derived FLAG-NS1/2 and FLAG-NS4 localized with NS7, a marker for the viral replication complex (Fig. S3C) (46). Having validated the use of FLAG-tagged viruses to study replication, we infected the BV2 microglial cell line with MNoV-NS1/2^FLAG^ and MNoV-NS4^FLAG^. Both FLAG-NS1/2 and FLAG-NS4 coprecipitated with VAPA but not NS7 or GAPDH (glyceraldehyde-3-phosphate dehydrogenase) (Fig. 3A, bottom). Thus, NS1/2, either independently or together with NS4, interacts with VAPA (45).

10.1128/mBio.00668-17.3 FIG S3  Supplemental material corresponding to Fig. 3. (A) Engineering infectious FLAG-tagged MNoV-CW1. The FLAG epitope tag was inserted into sites in NS1/2 and in NS4 (45). (B) Growth characterization of the NS1/2-FLAG (left) and NS4-FLAG (right) viruses. A multistep growth curve was performed by infecting BV2 cells with either virus at an MOI of 0.01 TCID_50_/cell. Virus was harvested at the specified time points, and viral titers were determined by TCID_50_ analysis. Two-way ANOVA and the Bonferroni posttest were performed for comparisons of the WT and mutant strains at each time point (*n* = 3). The limit of detection (LOD) was 11.2 TCID_50_/ml. Standard errors of the means (SEM) were calculated for each condition, but the SEM data do not appear on the graph in cases when the error bars lie within data symbol. (C) Immunofluorescence in BV2 cells infected with NS1/2-FLAG, NS4-FLAG, or WT virus. The MOI was 5 TCID_50_/cell (12 hpi). Dual staining was performed for FLAG and NS7. Download FIG S3, EPS file, 0.7 MB. Copyright © 2017 McCune et al. 2017 McCune et al. This content is distributed under the terms of the Creative Commons Attribution 4.0 International license.

To test for direct NS1/2-VAPA interaction independently of the presence of other viral proteins, we assessed NS1/2 interaction with VAPA by mammalian 2-hybrid (M2H) analysis. In this assay, interaction between a “bait” protein and a “prey” protein generates a luciferase signal. As previously reported (12, 23, 47), we detected VAPA interaction with itself, the host protein oxysterol-binding protein 1 (OSBP), and human norovirus NS1/2^GI^, validating use of M2H analysis as an approach to assess VAPA interactions (Fig. 3B). NS1/2^MNoV^ from either MNoV strain CW3 or MNoV strain CR6 interacted with VAPA (Fig. 3B). Of interest, NS1/2 also interacted with VAPB (Fig. 3B).

### NS1/2 interacts with FFAT-binding residues in VAPA MSP domain.

**FIG 4:**
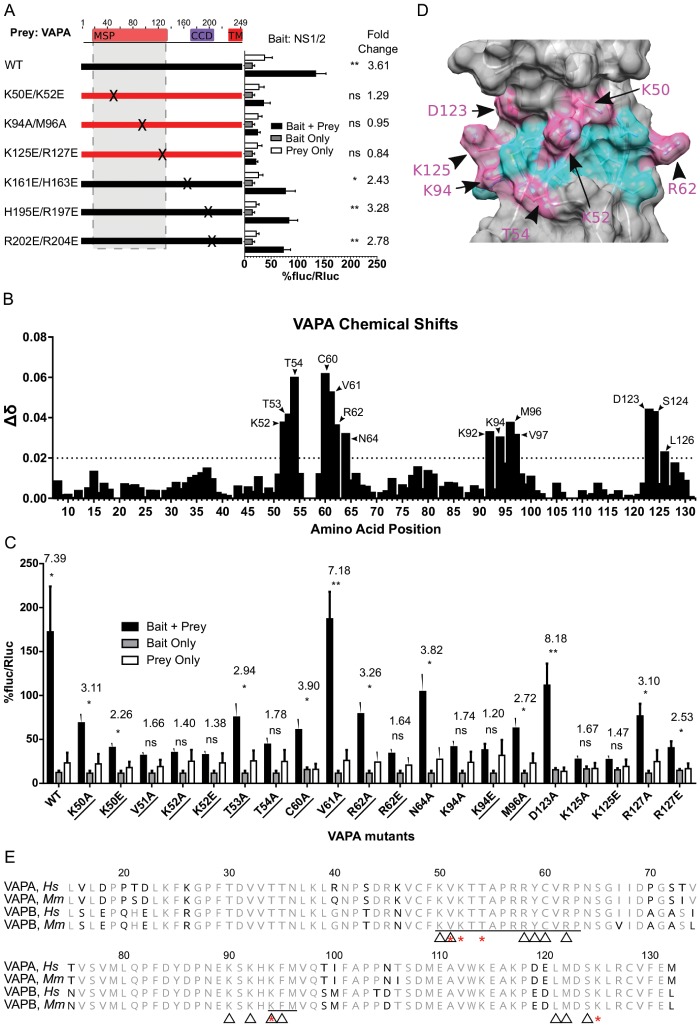
NS1/2 binds FFAT-interacting residues in MSP domain of VAPA. (A) M2H interaction of NS1/2^MNoV^ with VAPA mutants. (B) Chemical shift perturbations of amide resonances upon unlabeled-NS1^CW3^ titration into ^15^N-labeled VAPA MSP. The horizontal broken line represents the threshold. (C) M2H analysis of additional single-residue mutant VAPA. Designations of residues interacting with FFAT are underlined (one-way ANOVA, Dunnett posttest; fold change data are shown at the top; *n* = 3). (D) Murine VAPA MSP domain (PDB 2CRI). Pink highlighting indicates residues that disrupted the NS1/2-VAPA interaction in M2H analysis when mutated; mutations in cyan residues did not disrupt interaction. (E) Multiple alignment of VAPA and VAPB MSP domains from human (*Hs*) and mouse (*Mm*). Residues indicated with a black character differ from consensus data. Red asterisks mark residues necessary for interaction in M2H analysis, and triangles mark residues that shifted in NMR during NS1/2 titration.

Many VAPA protein-protein interactions occur between the VAPA MSP domain and host cell proteins containing FFAT motifs. Structure-function analyses of FFAT-VAPA interactions support a model in which FFAT motifs from VAPA client proteins rest within a groove present on the surface of the VAPA-MSP domain (24–26). Within this groove, VAPA residues K50, K52, K94, M96, and K125 are critical for interaction with FFAT motifs. To test if these residues also engage NS1/2, we introduced the following mutations into VAPA: K50E/K52E, K94A/M96A, and K125E/R127E (Fig. 4A). Each of these mutation pairs decreased VAPA interaction with NS1/2 (Fig. 4A) as measured by M2H analysis. To test if NS1/2 interacts with sets of positively charged residues elsewhere in VAPA, we mutated additional sites in VAPA selected to have the sequence (H/R/K)X(H/R/K). Mutations K161E/H163E, H195E/R197E, and R202E/R204E had no effect on the NS1/2-VAPA interaction (Fig. 4A). We conclude that the NS1/2 interaction specifically required positively charged residues within the VAPA MSP domain.

In work presented below, we found that the NS1 domain of NS1/2 is required for NS1/2-VAPA interactions. To map the physical interactions between NS1 and the VAPA-MSP domain, we used nuclear magnetic resonance (NMR) to analyze the chemical shift perturbations of the ^15^N-labeled VAPA-MSP domain (M8–M132 of VAPA) titrated with increasing amounts of unlabeled NS1 (S28–R114 of NS1/2). This analysis revealed interactions between NS1/2 and four groups of residues on VAPA (Fig. 4B; K52–T54, C60–N64, K92–V97, and D123–L126). These groups of residues all mapped to the FFAT binding groove on a positively charged surface of the MSP domain. Furthermore, the VAPA residues that bind NS1/2 coincide with the FFAT-motif interaction surface on the MSP domain (24, 25). Using the same experimental approach, we did not observe any interactions of NS1 with the isolated coiled-coil domain (P133–S226 of VAPA; data not shown).

We independently verified the role of the VAPA residues identified above in NS1/2-VAPA interactions using M2H analysis (Fig. 4A). To this end, we replaced selected amino acids in the VAPA MSP domain with either glutamate or alanine and tested for the interaction of these mutant molecules with NS1/2. No interaction was detected with glutamate or alanine substitutions at positions V51, K52, T54, K94, and K125 (Fig. 4C and D). No interaction occurred after mutation of R62 to glutamate, but an interaction was present with alanine at this site (Fig. 4C). At positions K50, T53, V61, N64, M96, and R127, however, we observed interaction after replacing those residues with either glutamate or alanine (Fig. 4C and D). Notably, the residues within the VAPA-MSP domain that are necessary for interaction with NS1 are conserved in VAPB (Fig. 4E).

### Residues 47 to 54 of murine norovirus NS1 are necessary for interaction with VAPA.

**FIG 5:**
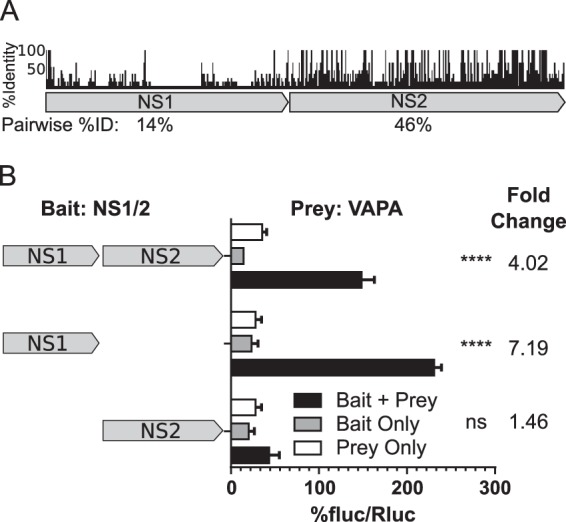
A poorly conserved NS1 domain within NS1/2^MNoV^ interacts with VAPA. (A) Alignment of NS1/2 from representative strains from each norovirus genogroup. %ID, percent identity. (B) M2H analysis of full-length or domain truncations of NS1/2^MNoV^ (CR6) with VAPA (one-way ANOVA and Dunnett posttest; fold change data are shown at the top; *n* = 3).

**FIG 6:**
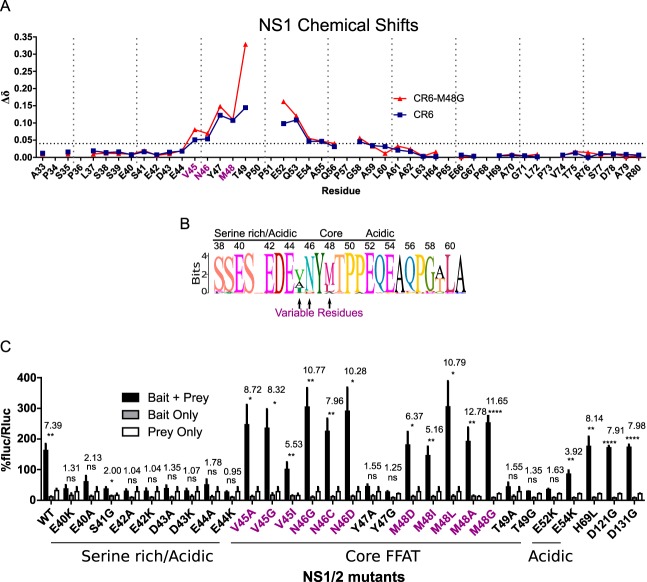
The N-terminal segment of NS1-MNoV interacts with VAPA. (A) Chemical shift perturbations of amide resonances upon titration of unlabeled VAPA into ^15^N-labeled NS1-CR6 and CR6^M48G^. The horizontal broken line represents the threshold. Purple residues are indicated as described for panel B. (B) Sequence logo of FFAT-like amino acid sequence of NS1/2 derived from BLAST alignment (Fig. S4C). The font size for each amino acid is proportional to percent conservation at each position. Residues exhibiting greater variability across MNoV strains are highlighted with arrows (colored purple here). (C) M2H interaction with NS1/2 substitutions (NS1/2, bait, VAPA, prey). Residues 69, 121, and 131 are not predicted to interact with VAPA. Purple residues are indicated as described for panel B (one-way ANOVA, Dunnett posttest; fold change data are shown at the top; *n* = 3).

While the NS2 domain is well conserved within the *norovirus* genus, NS1 is not (Fig. 5A). Accordingly, we predicted that the conserved NS2 domain contributed to the NS1/2 interaction with VAPA. Surprisingly, the MNoV NS1 domain containing residues 1 to 131 was sufficient to interact with VAPA whereas the NS2 domain did not interact (Fig. 5B). To define the specific NS1 residues interacting with VAPA-MSP, we analyzed the chemical shift perturbations of the NMR spectra of ^15^N-labeled NS1 (S28–R114 of NS1/2) with increasing amounts of unlabeled VAPA (M8–S226 of VAPA). The largest perturbations in NS1 from both the CR6 and CW3 strains of MNoV were observed for a core of interacting residues centered on Y47–Q53 (YMTPPEQ) (Fig. 6A and Fig. S4A). A longer sequence, encompassing residues I45 to A61, showed consistent but smaller perturbations (Fig. 6A and Fig. S4A). There are no observable amides in prolines; hence, no data were available for P50, P51, and P57.

10.1128/mBio.00668-17.4 FIG S4  Supplemental material corresponding to Fig. 6. (A) A portion of superimposed ^1^H-^15^N HSQC spectra of NS1^CW3^ with increasing levels of VAPA. NS1/VAPA molar ratios were as follows: 1:0.0 (red); 1:0.3 (orange); 1:0.6 (yellow); 1:1.2 (green); 1:2.6 (cyan); 1:4.9 (blue). Assignments and peak positions are shown for free NS1 sample. The inset shows the structure of NS1 28–114 (PDB 2MCH), with core VAPA interacting residues labeled in blue. (B) Chemical shift perturbations of amide resonances upon unlabeled-VAPA titration into ^15^N-labeled NS1-CW3, CW3^T49G^, and CW3^E52K^. The horizontal broken line represents the threshold. (C) BLAST alignment of NS1/2 sequence resembling FFAT. The bottom two sequences are from rat norovirus, representing the most divergent NS1/2 sequence within GV. Download FIG S4, EPS file, 7.9 MB. Copyright © 2017 McCune et al. 2017 McCune et al. This content is distributed under the terms of the Creative Commons Attribution 4.0 International license.

To test the importance of this core of interacting residues, we carried out experiments with three mutant forms of NS1, namely, NS1-CR6^M48G^, CW3^T49G^, and CW3^E52K^. The heteronuclear single-quantum coherence (HSQC) spectra obtained for the mutants were similar, indicating that these mutations did not destabilize tertiary structures (data not shown). NS1-CW3^T49G^ and CW3^E52K^ mutations decreased binding to VAPA to undetectable levels, while NS1-CR6^M48G^ interacted with VAPA (Fig. 6A and Fig. S4B). Within the NS1 domain, the VAPA interacting residues are predominantly within the segment K26–P57, which shows a highly dynamic conformation in isolated NS1 (10). The last few interacting residues of the core residues of NS1 that interact with VAPA are in the structured domain of NS1 (G58–R114) (10).

### Murine norovirus NS1 contains a mimic of host FFAT domains.

The FFAT motif is responsible for interactions of host proteins with the MSP domain of VAPA. We identified residues 40 to 54 as the domain of NS1 which interacts with the MSP domain of VAPA. Thus, we compared this region of NS1 with FFAT motifs. Generally, FFAT motifs contain a core bulky aromatic residue flanked by acidic residues (22, 27). Correspondingly, residues 40 to 54 of NS1/2 contain a bulky aromatic (Y47) flanked by acidic residues E40, E42, D43, E44, E52, and E54 (Fig. 6B). Interestingly, this sequence is conserved across MNoV strains (Fig. 6B and Fig. S4C), though positions 45, 46, and 48 are variable. The strong conservation of certain amino acids in this region suggested that this motif has functional importance.

To test which residues within this domain contribute to interaction with VAPA, we introduced single-residue mutations and assessed their effect by M2H analysis. For positions in the N-terminal acidic segment, mutations E40A, E40K, E42A, E42K, D43A, D43K, E44A, and E44K blocked NS1/2-VAPA interactions, while S41G maintained a detectable interaction (Fig. 6C). Within the FFAT-like core segment, Y47A, Y47G, T49A, and T49G ablated NS1/2 interactions with VAPA. Residues at positions 45, 46, and 48 are variable across MNoV strains (Fig. 6B and Fig. S4C). To test the function of amino acids in these positions, we introduced variants observed in other MNoV strains, including V45A, V45I, N46C, N46D, M48A, and M48L, as well as variants not observed in MNoV isolates, including V45G, N46G, M48D, M48I, and M48G. Mutations at these positions did not disrupt interactions, suggesting that the interaction is preserved among variable sequences in these positions across strains (Fig. 6C). For C-terminal acidic residues, E52K mutation disrupted the interaction, but E54K maintained the interaction. Additionally, mutations outside this region, including H69L, D121G, and D131G, did not prevent interaction (Fig. 6C).

In summary, the S40-E54 region of NS1 mimics the host FFAT motif and serves as the basis for interaction with the VAPA MSP domain based on the following findings: (i) the order and chemical nature of the amino acids mimic those of the FFAT motif (acidic, bulky aromatic, and then acidic); (ii) each of those acidic or bulky aromatic amino acids is necessary for binding VAPA; (iii) this NS1 region interacts with the same region of VAPA which binds to FFAT motifs in host proteins; and (iv) these critical amino acids are conserved across norovirus strains.

### NS1/2-VAPA interactions are required for recovery of murine norovirus from infectious clones.

**FIG 7:**
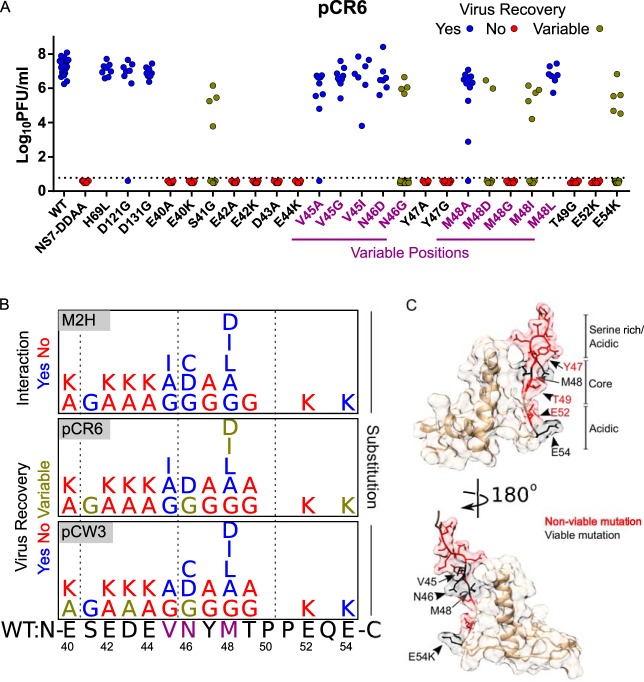
NS1/2 interaction with VAPA enhances recovery of murine norovirus from infectious clones. (A) Recovery titers of mutants of MNoV strain pCR6. Data represent passage 1 titers (*n* = 7 to 20). (B) Summary of interaction of NS1/2 mutants with VAPA in M2H analysis, and recovery of virus from infectious clones for CW3 and CR6 NS1/2 mutants. (C) Molecular surface-and-ribbon diagram of solution structure of NS1-MNoV (PDB 2MCD [10]) with viable (black) and nonviable (red) mutants.

We used an infectious molecular clone of MNoV to introduce mutations and to determine the importance of the NS1/2-VAPA interaction and the specific amino acids in the NS1/2 FFAT-like domain required for MNoV growth. Mutations were introduced in a plasmid encoding the CR6 viral genome, and recovery of infectious virus was assessed after transfection of the plasmid into permissive cells. We noticed three patterns of recovery of infectious virus in these experiments (Fig. 7A): (i) some NS1/2 mutations had no discernible effect on virus recovery (V45G, V45A, V45I, N46D, M48A, M48L, H69L, D121G, and D131G); (ii) some NS1/2 mutations resulted in variable recovery (S41G, N46G, M48I, M48D, and E54K); (iii) some NS1/2 mutations completely eliminated virus recovery (E40A, E40K, E42A, E42K, D43A, D43K, E44A, E44K, Y47G, Y47A, M48G, T49G, T49A, and E52K). We saw similar patterns of virus recovery after insertion of mutations into NS1/2 in the CW3 genome, with the following exceptions: NS1/2 mutations S41G, N46C, M48I, M48D, and E54K resulted in consistent recovery of virus; E40A and D43A mutations resulted in variable virus recovery; I45G mutation completely prevented virus recovery (Fig. S5).

10.1128/mBio.00668-17.5 FIG S5  Supplemental material corresponding to Fig. 7. Data represent recovery titers of mutants of MNoV strain pCW3. The figure presents passage 1 titers (*n* = 7 to 20). Download FIG S5, EPS file, 0.3 MB. Copyright © 2017 McCune et al. 2017 McCune et al. This content is distributed under the terms of the Creative Commons Attribution 4.0 International license.

Importantly, this mutational analysis of the NS1 domains of two strains of MNoV revealed a strong correlation between mutations that perturbed VAPA interaction (Fig. 7B, top panel) and those which diminished recovery of virus (Fig. 7B, bottom two panels). Side chains for residues that were critical for recovery of virus primarily mapped to a sequence showing highly dynamic behavior in free NS1 and a few N-terminal residues of the NS1 structured domain (10) (Fig. 7C). The specificity of the relationship between side chain and function within this region is strikingly revealed by comparing the role of the tyrosine at position 47, which was important for virus recovery, and the immediately adjacent methionine at position 48, where multiple amino acid substitutions were tolerated.

## DISCUSSION

In this report, we define the importance of the VAPA host protein and its interaction with viral nonstructural protein NS1/2 in replication of MNoV. We confirmed the previously identified interaction between a human norovirus NS1/2 protein and VAPA (12) and found that this interaction is shared with the NS1/2 proteins of two MNoV strains. Using a variety of approaches, including analysis of the interaction of the proteins *in vitro* and in cells, we delineated the structural basis for the interaction between VAPA and NS1/2 and used these data to test for the importance of specific amino acids in NS1/2 for viral replication and for the interaction between VAPA and NS1/2. These studies support the concept that VAPA is a proviral host protein for MNoV infection and that interaction between NS1/2 and VAPA is important for viral replication. Remarkably, the MNoV NS1 domains appear to mimic host VAPA-binding proteins through the conservation of a region that mimics host FFAT domains present in VAPA MSP domain-interacting proteins.

### Norovirus mimicry of host FFAT motifs.

Mimicry of host molecules and motifs is a pervasive evolutionary theme enabling microbes to hijack host processes (48). While efforts have been made to predict mimicry on a large scale (49), detecting structural and/or functional domain mimics requires validation through detailed studies of individual microbial molecules. Other microbial proteins involved in targeting VAPA mimicry via a FFAT motif have not been reported. It will be interesting to determine whether FFAT domain mimicry is a common strategy for microbial proteins that target VAPA. If so, small molecules that target this interaction surface may have antiviral or antimicrobial properties for multiple microbes that similarly bind VAPA. In this regard, it is important that FFAT motifs tolerate variation at many positions (22, 27), are relatively short, and are unstructured in solution (24), potentially enabling viruses or other organisms to evolve strategies to target VAPA. It is interesting that much of the region of MNoV NS1/2 that contains the FFAT mimic is unstructured in the purified NS1 domain (10). It seems possible that the interaction of these domains with the MSP domain of VAPA is somehow enhanced by the unstructured nature of this region.

The greatest similarity of the MNoV NS1/2 sequences to host FFAT motifs was identified in the N-terminal and C-terminal portions of the motif. The core sequence was less similar, notably lacking a phenylalanine followed by D/E and instead encoding a tyrosine without a flanking acidic residue. The third position of host FFAT motifs (the second of the two F residues, which define the motif in host proteins) tolerates a wide range of residue substitutions without loss of function. Similarly, both NMR experiments and M2H experiments performed with the NS1 M48G mutant have shown consistent tolerance of variability at this site. Nonetheless, at the structural level, the mode of binding mode of NS1/2 to VAPA showed remarkable similarity to the binding of host FFAT motifs to VAPA, for example, by interaction with specific VAPA amino acids in the MSP domain. It is therefore interesting that the core portions of host and norovirus FFAT motifs differ in some regards, suggesting that there may be specific properties of the interaction that are unique to the viral FFAT motif. Future examination of the molecular basis of the interaction between human norovirus NS1/2 and VAPA and of the conservation of relevant amino acids across norovirus genogroups and strains will be useful and interesting.

### Role of VAPA in norovirus replication.

Importantly, while we studied VAPA in detail, we also found that the related VAPB protein plays a role in MNoV replication and binds NS1/2. It seems possible that these two proteins play similar roles in NoV replication. For VAPA, it is clear that the stages of viral replication after entry and before minus-sense viral RNA synthesis are affected by VAPA. Nevertheless, our work did not reveal the mechanism by which VAPA participates in the viral life cycle. We have considered two possibilities (not mutually exclusive) for the function of the NS1/2-VAPA interaction at this early stage of viral replication. First, the NS1/2-VAPA interaction could localize NS1/2 to the ER in order to initiate formation of the membranous viral replication compartment. Notably, the advantage afforded by direct interactions of viral proteins with VAPA and VAPB proteins has been reported for hepatitis C virus (28, 29), which also required rearrangements of intracellular membranes to create a replication complex. MNoV NS1/2 is associated with the ER when expressed independently of other viral proteins (46, 50), and VAPA is an ER-resident protein, suggesting the possibility of a role for VAPA in NS1/2 localization. It is notable that the NS1 domain that contains the FFAT motif mimic would be the first portion of the polyprotein synthesized from viral plus-sense RNA and might therefore contribute to coordination of initial steps of viral replication at the ER prior to synthesis and processing of the rest of the viral polyprotein.

Second, it is also conceivable that the interaction of NS1/2 with VAPA alters lipid metabolism through competition for the interactions between VAPA and VAPA client proteins that also have FFAT domains. In this regard, it is not known whether any of the specific processes carried out by VAPA client proteins are important for enhancing or inhibiting norovirus replication. The methods required to address this issue are likely to be complex, since VAPA interacts with multiple client proteins such as OSBP and ceramide transfer protein (CERT) and is involved in a range of processes in the cell, including nonvesicular lipid transfer (20, 23, 51) and lipid metabolism (51, 52), and is present at membrane contact sites (53–57). Nevertheless, the conservation of a structural motif related to the FFAT motifs found in proteins that interact with the MSP domain of VAPA indicates the value of dissecting the possible role of VAPA-dependent functions in the viral life cycle and the impact of NS1/2 function on VAPA-dependent proteins.

## MATERIALS AND METHODS

### Cells and media.

293T, BV2, and RAW 264.7 cells were maintained in Dulbecco’s modified Eagle’s medium (DMEM) with 10% fetal calf serum (FBS), 1% penicillin/streptomycin (Pen/Strep), 2 mM l-glutamine, and 10 mM HEPES. All transfections were performed with TransIT-LT1 (Mirus) unless otherwise noted. The Genome Engineering and iPSC Center (St. Louis, MO) engineered *Vapa*^*−/−*^ RAW 264.7 cell lines 1E6 and 3A11. Briefly, guide RNAs (5′ GGCGAAGCACGAGCAGATCCTGG 3′ and 5′ GATCTGCTCGTGCTTCGCCATGG 3′) targeting *Vapa* were electroporated into RAW 264.7 cells transiently expressing Cas9. Cells were clonally selected and verified for disruption of the endogenous locus via the Cel-1 nuclease assay and were then subjected to deep sequencing to identify frameshift mutations.

### Molecular cloning.

NS1/2 from strain MNoV CR6 and CW3 infectious clones (11) and GI (NC_001959), as well as VAPA (NM_013933), were cloned into Gateway vector pDONR221 (Life Technologies, Inc.) and subcloned using Gateway destination vectors, including a modified phage-FLAG-hemagglutinin (HA)-attR1-ccdB-attR2-internal ribosome entry site (IRES)-PuroR lentiviral construct. Cloning of mutant MNoV plasmids (58) was done by site-directed mutagenesis using Q5/KLD mix or Phusion (New England Biolabs) as described in reference 59. The MNoV-NS1/2^FLAG^ infectious clone was generated similarly to MNoV-NS4^FLAG^ (45), with FLAG tag nucleotide sequence inserted after nucleotide 383.

### Virus reagents and procedures.

Stocks were generated as described previously (11). Briefly, infectious clones were transfected into 293T cells to produce infectious virus, which was passaged twice on RAW 264.7 cells. Clarified supernatant was subjected to ultracentrifugation, resuspended in phosphate-buffered saline (PBS), and quantitated by plaque assay. The recovery of infectious FLAG-tagged MNoV was described previously (60). Briefly, infectious clones were transfected in BSRT7 cells infected with fowlpox virus expressing T7 RNA polymerase. BV2 cells were inoculated with the recovered viruses, frozen/thawed upon appearance of cytopathic effects, centrifuged to remove cellular debris, and quantitated by 50% tissue culture infective dose (TCID_50_) analysis. The stability of FLAG tag insertions at passage 3 was verified by RT-PCR and sequencing of the viruses at relevant genomic locations and by immunoblotting against FLAG tags using infected lysates (data not shown). MNoV infectious clones with novel mutations were transfected in 293T cells as described above and passaged once on RAW 264.7 cells, and virus concentrations were assessed using plaque assay. For virus growth analysis, MNoV was inoculated at indicated multiplicities of infection (MOI) into cells in suspension for 30 min on ice and was subsequently washed three times with complete media and harvested at indicated times postinfection. Quantitation of norovirus by plaque assay was performed as described previously (11) except using adherent RAW 264.7 cells. TCID_50_ data were determined on BV2 cells as described previously (61). For viral RNA electroporations, RNA was isolated from norovirus stocks with TRIzol (Thermo Fisher) and transfected by the use of an Amaxa Mouse Macrophage Nucleofector kit (Lonza). Lentivirus was prepared as described previously (62), except transfections were performed with TransIT LT1 (Mirus), and cells were maintained in media with puromycin (5 μg/ml) 48 h after transducing.

### Flow cytometry.

Cells were infected as described above. At indicated times, supernatant was collected for determinations of viral titers and cells were prepared for fluorescence-activated cell sorter (FACS) analysis as described in reference 62, except using primary antibody anti-NS1/2 rabbit sera (V. Ward) (1:2,500), and data were acquired on an LSR II or FACSCalibur (BD Biosciences) flow cytometer. Analyses were performed using FlowJo (Treestar, OR).

### Confocal microscopy.

BV2 cells were seeded on glass coverslips and infected at an MOI of 1 TCID_50_/cell. At 12 hpi, cells were fixed with 4% paraformaldehyde (PFA)–PBS, quenched with 0.1 M glycine–PBS, and permeabilized with 0.2% Triton X-100–PBS. After blocking was performed (using PBS plus 0.1% Tween 20 [PBST] with 1% bovine serum albumin [BSA]–1% normal goat serum [Sigma-Aldrich]), cells were stained with mouse monoclonal anti-FLAG M2 antibodies (Sigma-Aldrich) and rabbit polyclonal anti-NS7 antibodies (1:1,000) at room temperature for 1 h, triply washed (PBST), and then stained with goat anti-mouse IgG Alexa Fluor 488 and goat anti-rabbit IgG Alexa Fluor 546 (1:1,000) at room temperature for 1 h. Coverslips were triply washed and then mounted with Mowiol medium containing DAPI (4′,6-diamidino-2-phenylindole) stain. The confocal images were taken using a Zeiss 510 Meta laser confocal microscope.

### Immunoprecipitation.

For anti-FLAG immunoprecipitation, BV2 cells were infected at an MOI of 10 TCID_50_/cell and were harvested 8 hpi. Cells were triply washed with cold PBS before lysis was performed with a mixture containing 50 mM Tris-HCl (pH 7.4), 150 mM NaCl, 1 mM EDTA, 2 mM MgCl_2_, 1% Triton X-100, 1% (vol/vol) protease inhibitor cocktail (Promega), and 0.1% Benzonase (Sigma-Aldrich). The lysates were incubated on ice for 30 min before being spun down for 10 min at 15,000 rpm at 4°C. The supernatants were collected, and the protein concentrations were determined by bicinchoninic acid (BCA) assay (Thermo Fisher). The anti-FLAG M2 affinity agarose gel (Sigma-Aldrich) was prewashed twice with TBS buffer (50 mM Tris-HCl [pH 7.5], 150 mM NaCl). A 2-mg volume of total protein in 1 ml lysis buffer was loaded onto 40 µl anti-FLAG agarose and incubated 4°C overnight with rotation. After removal of unbound protein by centrifugation at 5,000 × *g* for 30 s at 4°C and three more washes with TBS buffer, the bound proteins were eluted by addition of 50 µl of 2× SDS-PAGE sample buffer and heating at 95°C for 3 min.

### Western blotting.

Laemmeli buffer was added to samples and then boiled for 10 to 15 min. Protein was resolved on 10% SDS-PAGE Tris-glycine gels. Protein was transferred semidry to polyvinylidene difluoride (PVDF) membranes, blocked with 5% milk–Tris-buffered saline with Tween 20 (TBST), and then incubated with antibody overnight at 4°C. Membranes were triply washed with TBST and then incubated for an hour with horseradish peroxidase (HRP)-conjugated secondary antibody. Membranes were triply washed and incubated with ECL or ECL2 reagent (Pierce), and then signal was detected on film (MidSci). For densitometry, NS1/2 band density was calculated using ImageJ, normalized to GAPDH band density, and then reported as a ratio to the WT from each respective time point. We used the following antibodies: polyclonal rabbit NS1/2 antisera, a kind gift from Vernon Ward; anti-VAPA clone K-15 (sc-48698; Santa Cruz Biotechnology), anti-FLAG (M2; Sigma-Aldrich), and anti-HA (H9658; Sigma-Aldrich) (conjugated to HRP using a Lightning-Link HRP antibody labeling kit [701-0000; Innova Bioscience]); GAPDH-HRP (G9295-25UL; Sigma-Aldrich); anti-actin (A5316; Sigma-Aldrich); and anti-rabbit HRP (111-035-003), anti-goat HRP (705-035-003), and anti-mouse HRP (115-035-146) (Jackson ImmunoResearch, Inc.).

### Strand-specific qPCR.

Cells were infected as described above. At each time point postinfection, cells were lysed and total cellular RNA was extracted using a GenElute mammalian total RNA Miniprep kit (Sigma-Aldrich). Quantities of genomic positive/negative RNAs were determined using strand-specific real-time quantitative PCR (RT-qPCR) according to the method described in reference 44 with the following changes: 100-ng total RNA was used in each RT reaction, and 5 µl of cDNA was used for genomic negative qPCR. The mean of log_10_ genome equivalents (gEq) per nanogram of total RNA of mock-infected cells was used as the limit of detection (LOD). The results were obtained using a ViiA7 real-time PCR system.

### Assessment of VAPB in murine norovirus replication.

BV2 cells were transduced with lentivirus expressing Cas9 and blasticidin resistance and were maintained in 4 µg/ml blasticidin. Blasticidin-selected cells were then transduced with lentivirus expressing puromycin resistance and either with no single-guide RNA (sgRNA) (empty) or with sgRNA directed against CD300lf, Vapa, or Vapb. Cells were maintained in 4 µg/ml puromycin. Cells were infected at an MOI of 0.1 without washing and were incubated at 37°C for 18 h. Cells were fixed and prepared for FACS analysis as described above. Each point represents an independent MNoV infection; cells were derived from 3 independent transductions of sgRNA-expressing lentivirus. For percent nonhomologous end joining (%NHEJ) estimates, DNA was isolated from cells using QiaAMP (Qiagen), melted and annealed on a thermocycler, treated with T7 endonuclease at 37°C for 1 h, and resolved on 2% agarose gel. Fragment densities were quantified using ImageJ, and percent cleavage was calculated using the following formula: %NHEJ = 100 * [1 − (parental fraction)^1/2^], where parental fraction = (band intensity parental band)/(band intensity parental band + band intensity lower fragments).

### Mammalian 2-hybrid assays.

Checkmate vectors (Promega) pACT (prey) and pBIND (bait) were converted to Gateway destination vectors, and genes were subcloned using Gateway LR reactions (Life Technology). Subsequent M2H analysis was performed as described in reference 63. In brief, 7.5 fmol bait and prey plasmids were transfected with 100 ng pG5 plasmid into subconfluent 293T cells. At 48 to 51 h posttransfection, cells were lysed and luminescence was measured by the use of a dual-luciferase reporter assay (Promega) using an Opticomp II (MGM Instruments) luminometer. All data shown represent *n* = ≥3 and were analyzed by one-way analysis of variance (ANOVA) and the Tukey posttest, comparing the greater of the bait-only value and prey-only value to the value corresponding to the combination of the bait data plus the prey data. Fold change was calculated from the value representing the average of the combination of the bait data plus the prey data/the greater of the bait data and the prey data.

### Vapa mutant mouse.

The Washington University Animal Studies Committee approved all mouse studies performed here. Mice were bred and housed per university guidelines. Day 0.5 B6/J inbred embryos underwent pronuclear microinjection with gRNA and Cas9-mRNA, and then embryos were implanted in surrogate mothers as described previously (64). Mutations in live-born pups were identified by isolation of tail DNA, PCR amplification of the Vapa targeted locus, and Sanger sequencing. Genotypes were verified by TOPO-TA (Life Sciences) cloning of the amplicons and Sanger sequencing. Genotyping was performed as follows. For mutant line 1, primers were designed to amplify the Vapa locus (F-CTGCTGAGCGGACAGGCTG, R-CGCAAGATGGCGGCGGAG) (WT, 500 bp; deletion, 440 bp). For mutant line 2, genotyping to detect single-base-pair insertion was designed as described in reference 65. In brief, primers designed to detect specifically the WT (F-GGCCCCGTCCTAGAGCTCCG, R-ATATGATAGTAACTATCCAGGATCTGCTCGTGCTACGC) amplified a 180-bp product. Primers detecting the mutant (F-GGCCCCGTCCTAGAGCTCCG, R-AAAAACCAGGATCTGCTCGTGCTTAGG) amplified a 159-bp product. Genotyping was verified by Sanger sequencing.

### Protein preparation for NMR experiments.

Natural-abundance protein and the ^15^N-labeled N-terminally His_6_-tagged 28–114 domain of MNoV NS1/2 protein were purified as described previously (10). Three fragments of natural-abundance protein and ^15^N-labeled N-terminally His_6_-tagged murine VAPA protein were purified from *Escherichia coli* expression plasmids as follows. (i) The VAPA MSP domain (8–132) gave excellent NMR spectra. (ii) The MSP domain with linker and coiled-coil domain (8–226) gave excellent NMR spectra and formed stable dimers in solution confirmed by size exclusion chromatography and diffusion NMR experiments. (iii) A VAPA fragment (133–226) showed a dimeric size in solution and NMR spectra indicative of contributions from α-helical and disordered segments. Protein samples were concentrated and dialyzed extensively against 10 mM KH_2_PO_4_–20 mM KCl (pH 7.0). Final concentrations of NS1/2 28–114 (ε_280_ = 13,940 M^−1^ cm^−1^) and VAPA (ε_280_ = 8,250 M^−1^ cm^−1^) were 0.4 mM and 1.6 mM, respectively, as determined spectrophotometrically. All samples contained reducing reagent (1 mM dTCEP [deuterated tris(2-carboxyethyl)phosphine]—5% D_2_O) for the lock signal and 0.5 mM DSS for chemical shift reference.

### Chemical shift perturbation experiments.

After 24 h of dialysis against the same buffer solution, protein samples were mixed by stepwise addition of VAPA solution. Each addition was followed by NMR experiments, carried out at 25°C on a Bruker 600-MHz instrument equipped with a cryoprobe. First, for each ^15^N-labeled NS1/2 protein construct, ^15^N-^1^H HSQC spectrum was recorded for an NS1/2 protein only. Following that step, 5 to 6 spectra were recorded after each addition of natural-abundance VAPA, typically at 0.5-fold to 10.0-fold excess over the NS1/2 concentration present. NMR data were processed (Topspin 3.2; Bruker), and the chemical shift perturbations were analyzed using NMRFAM-SPARKY (66). The chemical shift assignments for NS1/2 (BMRB entries 19439 and 19444) and closely related human VAPA (BMRB entry 7025) are available in the Biological Magnetic Resonance Data Bank (BMRB). The specific values of chemical shifts for the buffer conditions and protein constructs used here were verified by acquisition of a standard suite of triple resonance experiments performed on ^13^C/^15^N-labeled samples. Chemical shift perturbations on ^15^N-labeled VAPA were analyzed in analogous fashion, except that the initial concentrations of VAPA and NS1/2 were 0.1 mM and 2.2 mM, respectively, with stepwise addition of NS1/2. Figures show combined differences of ^1^H and ^15^N chemical shifts observed between zero and the highest concentration of unlabeled protein used. The combined differences in units (in parts per million) were calculated as $\Delta \delta =\sqrt{\left\{\frac{1}{2}\left[{\left({\Delta \delta }_{H}\right)}^{2}\;+\;{\left(\frac{{\Delta \delta }_{N}}{5}\right)}^{2}\right]\right\}}$ and are referred to as chemical shift perturbations. The threshold for perturbations interpreted as specific protein-protein interactions was set at 4 standard deviations above the mean perturbation, excluding the highest perturbations for each data set.

### Statistics and software.

All statistics were calculated using GraphPad Prism (ns, *P* > 0.05; *, *P* ≤ 0.05; **, *P* ≤ 0.01; ***, *P* ≤ 0.001; ****, *P* ≤ 0.0001; all error bars signify standard errors of the means). Sequence alignments and analysis were performed in Geneious 9.1 (67). Molecular graphics were produced using UCSF Chimera (68).

### Accession number(s).

The chemical shift assignments for NS1/2 (BMRB entries 19439 and 19444) and closely related human VAPA (BMRB entry 7025) are available in the BMRB database.
